# Evidence for the Pathogenicity of a *CFH* Variant in a Multigenerational Family with Cuticular Drusen

**DOI:** 10.3390/medicina61091649

**Published:** 2025-09-11

**Authors:** Egle Preiksaitiene, Viktorija Gurskytė, Violeta Mikštienė, Rasa Strupaitė-Šileikienė, Ramūnas Dzindzalieta, Gunda Petraitytė, Justas Dapkūnas, Enrika Vyčaitė, Dovilė Karčiauskaitė, Linas Černiauskas, Algirdas Utkus

**Affiliations:** 1 Life Sciences Center, Vilnius University, 10257 Vilnius, Lithuania; 2 Faculty of Medicine, Institute of Biomedical Sciences, Vilnius University, 03101 Vilnius, Lithuania; 3 Center for Medical Genetics, Vilnius University Hospital Santaros Klinikos, 08406 Vilnius, Lithuania; 4 Center of Eye Diseases, Vilnius University Hospital Santaros Klinikos, 08406 Vilnius, Lithuania; 5 Faculty of Medicine, Institute of Clinical Medicine, Vilnius University, 03101 Vilnius, Lithuania; 6 Institute of Biotechnology, Life Sciences Center, Vilnius University, 10257 Vilnius, Lithuania

**Keywords:** cuticular drusen, early-onset drusen maculopathy, age-related macular degeneration, *CFH*, complement factor

## Abstract

*Background and Objectives:* Cuticular drusen are a rare form of early-onset drusen maculopathy, which falls within the spectrum of age-related macular degeneration. Previous research suggests that cuticular drusen can result from monogenic inheritance of pathogenic variants in the complement factor H coding *CFH* gene. These variants impair regulation of the alternative complement pathway, leading to increased central retinal inflammation, progressive tissue damage, and ultimately, vision loss. This study aims to assess the pathogenic potential of the variant NM_000186.4(*CFH*):c.1318C>T, previously classified as a variant of unknown significance. *Materials and Methods:* Eight individuals from three generations of a single family underwent ophthalmologic evaluation, including biomicroscopy, ophthalmoscopy, optical coherence tomography, and optical coherence tomography angiography. Subsequently, whole-exome sequencing of the proband‘s DNA sample was performed. Sanger sequencing was used to validate the whole-exome sequencing results and to assess segregation of the identified variant in the family. The individuals’ clinical, instrumental, and genetic data were collected and stored in the database iGENLIT. *Results:* the heterozygous NM_000186.4(*CFH*):c.1318C>T variant was detected in six family members. Of these, five have been clinically diagnosed with cuticular drusen. Three affected individuals are currently in their 40s and maintain good visual acuity. In two family members, the drusen progressed to choroidal neovascularization, fibrosis, and atrophy, resulting in profound visual loss at the ages of 54 and 62. One 21-year-old individual also carries the variant, but currently shows no evidence of drusen, likely due to age. *Conclusion:* In this study, we demonstrated a genotype–phenotype relationship in individuals with the NM_000186.4(*CFH*):c.1318C>T variant, which suggests its pathogenic role in the development of cuticular drusen and associated complications.

## 1. Introduction

Age-related macular degeneration (AMD) is the leading cause of irreversible visual impairment in older adults, causing a gradual loss of central vision and blindness in advanced stages [1,2,3]. The hallmark of AMD is the presence of drusen—extracellular material deposits underneath the retinal pigment epithelium (RPE), affecting the RPE cells’ function and altering the characteristics of the Bruch’s membrane [3,4].

Cuticular drusen (CD, MIM#126700) are a rare drusen subtype, causing early onset drusen maculopathy (EODM), which first appears in early adulthood and becomes symptomatic between 35 and 70 years [5,6,7]. When first described by Donald Gass in 1977, CD were considered a distinct clinical entity, characterized by numerous bilateral, symmetric, small (25–75 μm), round, yellow subretinal deposits in the macula and middle periphery [5,8,9]. However, more recent studies classify CD as a subtype of AMD based on shared pathological features—such as the location between the basal lamina (BL) of RPE and inner collagenous layer of Bruch’s membrane—as well as overlapping clinical and genetic findings [5].

AMD is a multifactorial disease. The most important risk factors are advanced age, cardiovascular disease, smoking, increased body mass index, and diet [10,11]. Genetic factors also significantly affect its pathogenesis, contributing up to 70% of the disease variability [12]. To date, more than 100 genetic loci have been associated with AMD. Most of them are common variants identified through genome-wide association studies and confer a polygenic mode of inheritance. The genes are involved in many cellular processes: complement pathway, cell metabolism, apoptosis, proliferation, motion and junction, angiogenesis, immune pathways, etc. [13]. A strong association between the complement factor H coding *CFH* gene variant p.(Tyr402His) and the risk of developing AMD has been observed—this polymorphism accounts for the majority of AMD risk and is found in 48–70% of patients with CD [11,14].

Mendelian inheritance is also a proven pathogenic mechanism of AMD, particularly in EODM patients. Boon et al. demonstrated that CD in young adults can result from monogenic inheritance of *CFH* pathogenic variants [11]. Duvvari et al. found rare *CFH* variants in 8.8% of CD cases [15]. Several other genes involved in the complement pathway, including *CFI* (MIM#217030) and *C3* (MIM#120700), have also been implicated in the development of the disorder. Their pathogenic variants disrupt complement cascade regulation, leading to excessive inflammatory response, a significant pathological mechanism in AMD [4]. Additionally, *EFEMP1* (MIM#126600) and *ARMS2/HTRA1* locus on 10q26.13 (MIM#611313 and MIM#602194) are listed as hereditary causes of macular drusen [4,16].

In this study, we present a family with five members diagnosed with EODM. These individuals carry a rare, previously unreported heterozygous variant in the *CFH* gene, further elucidating the genotype–phenotype relationship in EODM.

## 2. Materials and Methods

### 2.1. Bioethics and Samples

Research was conducted as part of the execution of Project “Mission-driven Implementation of Science and Innovation Programmes” (No. 02-002-P-0001) “Safer and Smaller gene Editing tools for CURing genetic diseases of the Eye in vivo (SECURE)”, funded by the Economic Revitalization and Resilience Enhancement Plan “New Generation Lithuania”. An approval to conduct biomedical research (No. 2024/5-1588-1043) was issued by the Vilnius Regional Biomedical Research Ethics Committee (Vilnius, Lithuania).

### 2.2. Ophthalmic Investigation

Ophthalmological testing was performed at VUHSK from 2016 to 2025. All available family members underwent the measurement of best corrected visual acuity (BCVA), slit lamp biomicroscopy, dilated fundus examination (DFE), optical coherence tomography (Heidelberg Spectralis^®^ OCT (Heidelberg, Germany) or Optovue Avanti^®^ Widefield OCT (Fremont, CA, USA)), and OCT angiography (AngioVue^®^ OCT Angiography (Fremont, CA, USA)).

### 2.3. DNA Extraction

gDNA was isolated from peripheral blood leukocytes of the proband and her relatives using the phenol-chloroform-isoamyl alcohol extraction method or QIAamp DNA Blood Midi Kit (Qiagen, Germantown, MD, USA) according to the manufacturer’s protocol [17].

### 2.4. Whole-Exome Sequencing

Whole-exome sequencing (WES) was performed by CeGaT (Tübingen, Germany) to sequence the DNA sample of the proband. Sequencing libraries were created according to the manufacturer’s guidelines using the Twist Human Core Exome Kit, the RefSeq, and the Mitochondrial Panel (Twist Bioscience, San Francisco, CA, USA). The high-throughput next-generation NovaSeq 6000 (Illumina Inc., San Diego, CA, USA) platform was used for sequencing with 2 × 100 base pairs (bp) read length. WES data were processed with Illumina bcl2fastq (2.20). Adapters were trimmed with Skewer (version 0.2.2) [18].

Subsequent data processing and analysis were performed at VUHSK. The raw sequences were aligned to the human reference genome (GRCh37/hg19) using the Burrows-Wheeler Aligner (BWA-mem version 0.7.17) [19]. The data annotation was made using the ANNOVAR (v.2020Jun08) [20]. Guidelines and recommendations provided by the American College of Human Genetics and Genomics (ACMG) [21], in silico tools and databases provided by ANNOVAR (e.g., SIFT, Polyphen2, GERP++, CADD, ExAC, GnomAD, 1000 Genome Project data, NCBI dbSNP, NCBI ClinVar), and the scientific literature were used to assess the pathogenicity of detected variants. Virtual ophthalmic gene panel (Table S1) filters were applied using a custom-built in-house script at Vilnius University Hospital Santaros Clinics. The candidate genome variants were checked and validated by the Integrative Genomics Viewer (IGV) visualization tool [22].

### 2.5. Sanger Sequencing

Sanger sequencing was performed to validate the WES data and assess the family’s segregation. Polymerase chain reaction (PCR) of sequences flanking the possibly disease-causative variant of the *CFH* gene was performed on gDNA samples of the proband and her relatives using specific primers (Table S2) designed with the Primer Blast tool 2.6.0 [23]. PCR was performed using Phusion High-Fidelity PCR Master Mix (Thermo Fisher Scientific, Waltham, MA, USA). PCR products were fractioned by 1.5% agarose gel (TopVision, Thermo Fisher Scientific, USA) electrophoresis and visualized under ultraviolet light.

Sequence analysis of the PCR product was carried out with BigDye^®^ Terminator v3.1 Cycle Sequencing Kit (Thermo Fisher Scientific, USA) on a SeqStudio 8 Flex Genetic Analyzer (Thermo Fisher Scientific, USA). The resulting sequences were aligned with the reference sequence of *CFH* (NCBI: NM_000186.4).

### 2.6. In Silico Protein Analysis

The amino acid sequence for the *CFH* protein was retrieved from the UniProt database under accession number P08603 (Consortium 2025). The structure model for the wild-type *CFH* protein was obtained from the AlphaFold Protein Structure Database [24]. The corresponding mutant protein structure was predicted using AlphaFold2 as implemented in ColabFold v1.5.5 [25,26]. The quality of the generated structure models was then assessed using the VoroMQA server [27]. Interatomic contacts within the quality-assessed structures were analyzed using the VoroContacts server [28], and potential protein-protein interactions were evaluated with the PPI3D server [29]. The evolutionary conservation of amino acid residues was calculated and visualized on the protein structure using the ConSurf server and database [30,31]. Finally, the predicted pathogenic impact of the variant was assessed using the AlphaMissense database [32].

### 2.7. Enzyme-Linked Immunosorbent Assay (ELISA)

The concentration of *CFH* protein in venous blood plasma samples was measured to assess the impact of the *CFH* variant on the concentrations of *CFH* protein in venous blood plasma and membrane attack complex in serum, respectively. Venous blood plasma samples of the family members were stored at −80 °C until analysis. Before assay, frozen samples were thawed at room temperature and vortexed. Complement regulatory Factor H levels in plasma samples were measured using a commercial ELISA kit (Elabscience^®^, Houston, TX, USA, E-EL-H6057) according to the manufacturer’s instructions. Plasma samples were diluted 1:10,000. Each well’s optical density (OD) was evaluated at 450 nm in a Multiskan SkyHigh microplate spectrophotometer (Life Technologies Holdings Pte Ltd., Singapore). All samples were assessed in duplicate. The concentration of Factor H in each sample was calculated from the optical density (OD) value using a calibration curve equation. This equation was derived from the OD values and corresponding concentrations of standard samples.

The concentration of sC5b-9 in serum samples was analysed using diagnostic ELISA kits as routine testing at VUHSK. The cutoffs provided by the manufacturer were used to assess the impact of the *CFH* variant on the concentration of membrane attack complex in serum samples of adult family members.

### 2.8. Statistical Analysis

Descriptive statistics was calculated for parametric variables, including means, standard deviations, and minimum and maximum values. Effect size (Hedges’ g) was calculated to assess the difference in sC5b-9 concentration of variant carriers vs. the defined normal range and plasma *CFH* concentration of *CFH* gene variant carriers vs. non-carriers. No formal statistical test was performed due to the limited sample size. All analyses were performed using R Statistical Software (version 4.4.1, R Core Team 2024) [33].

### 2.9. Data Management

The clinical, instrumental, and genetic data of the proband and family members were collected and stored in the database iGENLIT created during implementation of the SECURE project.

## 3. Results

### 3.1. Clinical Presentation

**Figure 1:**
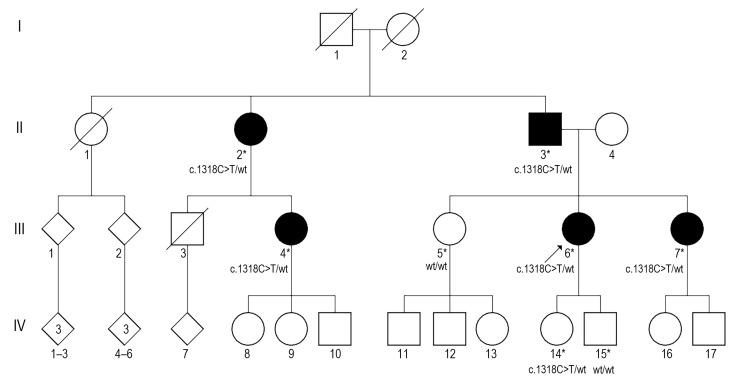
Genealogy of the family. An arrow shows a proband. Solid symbols indicate affected individuals. Tested individuals are indicated with an asterisk. Squares represent males, circles represent females, and diamonds indicate unspecified gender. Slashed symbols denote deceased family members. The genotypes are shown below the symbol.

We present eight individuals from three generations of a single family (Figure 1). The data illustrate the varying clinical characteristics of affected individuals depending on their age and the presence of the identified heterozygous *CFH* variant. Autosomal dominant inheritance was most likely.

**Figure 2:**
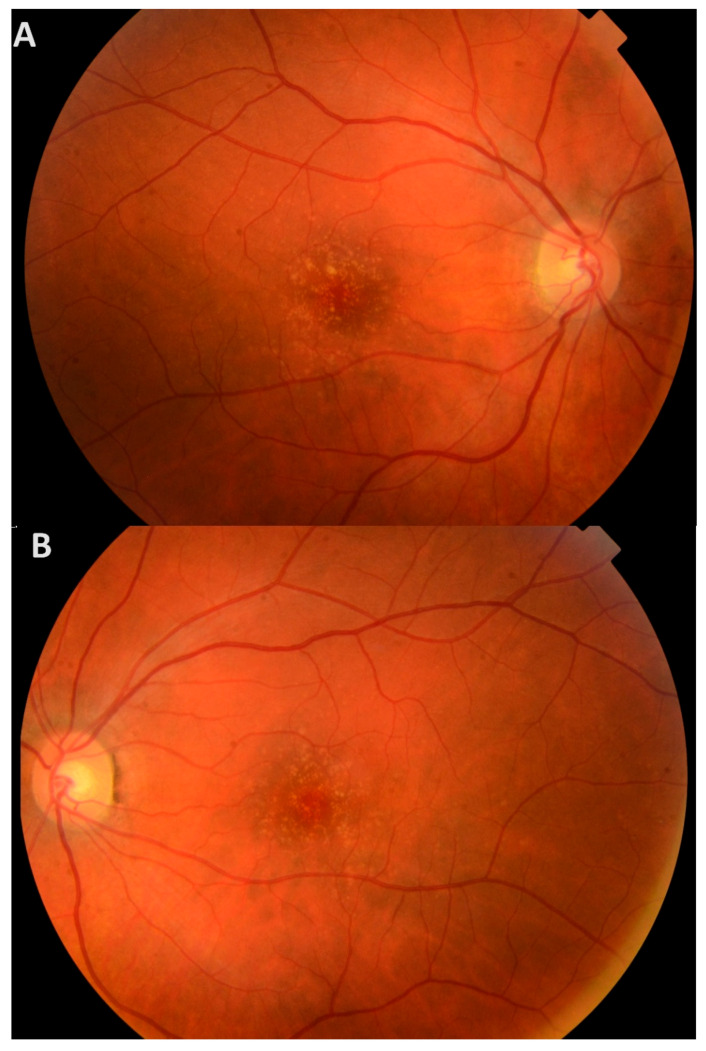
Fundus photographs of the proband’s right eye (**A**) and left eye (**B**) show numerous small, hard, yellow drusen in the posterior poles.

**Figure 3:**
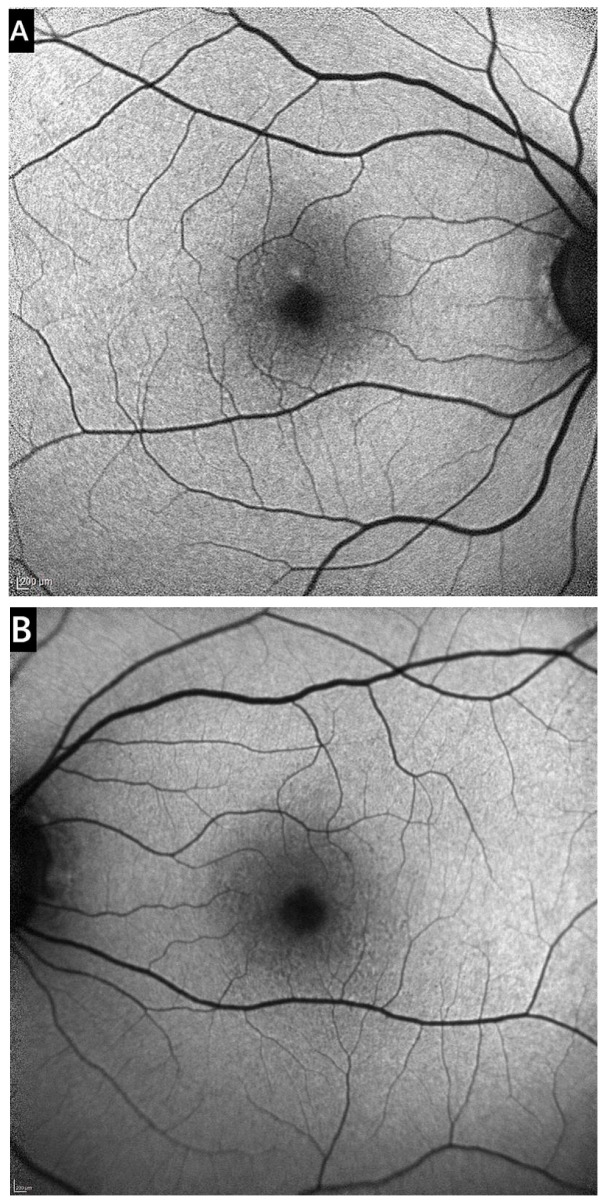
Blue-light fundus autofluorescence images of the proband’s right eye (**A**) and left eye (**B**). Some CDs appear to have hypoautofluorescent lesions with a hyperautofluorescent rim, while others display only hyperautofluorescence.

The proband (III-6) is an asymptomatic woman referred to our Center of Eye Diseases due to macular lesions found during a prophylactic check-up at 39 years of age. Her BCVA of both eyes was 20/16. During DFE, multiple small (40–88 µm in diameter) bilateral hard drusen and mild depigmentation were observed in the maculae (Figure 2). On blue-light fundus autofluorescence, the drusen appeared either hyperautofluorescent or hypoautofluorescent in the center, with a hyperautofluorescent rim (Figure 3). Macular OCT showed small RPE elevations arranged in a ‘saw-tooth’ pattern, suggesting CD. There was no evidence of choroidal neovascularization (CNV), intraretinal (IRF), or subretinal fluid (SRF) (Figure S1A,B). The proband has been monitored at our clinic for 9 years, and while the number of drusen has slightly increased, no complications have occurred. The participant has recently reported subjective vision worsening, and her current BCVA is 20/20.

The proband‘s father (II-3) complained of worsening vision at the age of 62. His BCVA of the right eye (RE) was 20/40, left eye (LE) 20/200. DFE and OCT revealed multiple small and intermediate confluent macular drusen. Additionally, subfoveal serous RPE detachments, CNV, SRF, and IRF were observed in OCT scans (Figure S1C,D). The father was diagnosed with neovascular AMD (nAMD), and treatment with intravitreal vascular endothelial growth factor inhibitors (anti-VEGF) was initiated. Partial reabsorption of IRF and complete reabsorption of SRF have been achieved. However, macular atrophy and fibrotic scars remain the cause of poor BCVA, which is currently 20/50 in the RE and 20/400 in the LE.

The proband’s paternal aunt (II-2) reported worsening vision at the age of 54. At 57, her BCVA of the RE was 20/40, LE–20/2000. She was also diagnosed with nAMD due to macular drusen, hemorrhages, CNV, and macular edema. Upon diagnosis, the LE already exhibited a fibrotic scar in the macula. Therefore, only the RE received intravitreal anti-VEGF treatment. The treatment was discontinued at 60 due to progression to chorioretinal atrophy (Figure S1E,F). Currently, the individual’s BCVA is 20/2000 in both eyes.

The proband has two asymptomatic sisters. The younger 38-year-old sister′s (III-7) BCVA of both eyes was 20/20. She had bilateral macular CD extending to the mid-periphery. However, the drusen were less numerous than in the proband. The drusen are evident in OCT scans as slightly raised nodules below the RPE and have not significantly progressed over the last 9 years (Figure S1G,H).

The proband’s 40-year-old sister′s (III-5) BCVA of both eyes was 20/16. DFE and OCT scans (Figure S1I,J) revealed only three small drusen in the superotemporal portion of the RE’s macula. The LE was unaffected. The drusen have not progressed over the last 7 years.

The proband’s 21-year-old daughter (IV-14) and 10-year-old son (IV-15) report no visual symptoms. Both children’s BCVA is 20/20 in both eyes, and no signs of CD have been observed (Figure S1K–N).

The proband’s paternal cousin (III-4) reported visual decline at the age of 46 years. Her BCVA was 20/30 in both eyes. DFE revealed multiple small, hard drusen in the maculae of both eyes. OCT scans confirmed a CD pattern with no associated complications (Figure S1O,P).

### 3.2. WES and Sanger Sequencing

WES revealed *CFH* (MIM#134370) heterozygous variant NM_000186.4(*CFH*):c.1318C>T, NP_000177.2:p.(Pro440Ser), rs761518362. The variant was evaluated as a variant of unknown significance in Varsome platform (3 points) (PM1 supporting (1 point): UniProt protein CFAH_HUMAN domain ‘Sushi 7’ has 25 missense/in-frame variants (4 pathogenic variants, 21 uncertain variants and no benign), which qualifies as supporting pathogenic); PM2 supporting (1 point): variant not found in gnomAD genomes, GnomAD exomes homozygous allele count = 0 is less than 2 for AD/AR gene *CFH*; PP3 Supporting (1 point): MetaRNN = 0.824 is between 0.748 and 0.841 ⇒ supporting pathogenic). Franklin Genoox tool classified the variant as a variant of unknown significance/likely benign (PM2 pathogenic moderate: extremely low frequency in gnomAD population databases (gnomAD maximal nonfounder subpopulations frequency: <0.001%, gnomAD maximal founder subpopulations frequency: 0.0%)). Furthermore, REVEL (score 0.35) and SIFT (score 0.007) assess the variant as uncertain, PROVEAN (score −6.3) classifies it as pathogenic, although PrimateAI (score 0.34) and PolyPhen-2 (score 0.41) predict it to be benign. CADD score for the variant is 22. The variant has not been described in scientific literature, and functional analysis has not been performed.

Segregation analysis showed the variant was inherited from the affected father (II-3). It was also identified in gDNA samples of affected relatives: the proband’s daughter (IV-14), sister (III-7), paternal aunt (II-2), and cousin (III-4). Based on these data, we classify the alternative allele of the *CFH* gene as a likely pathogenic variant.

### 3.3. Computational Analysis

**Figure 4:**
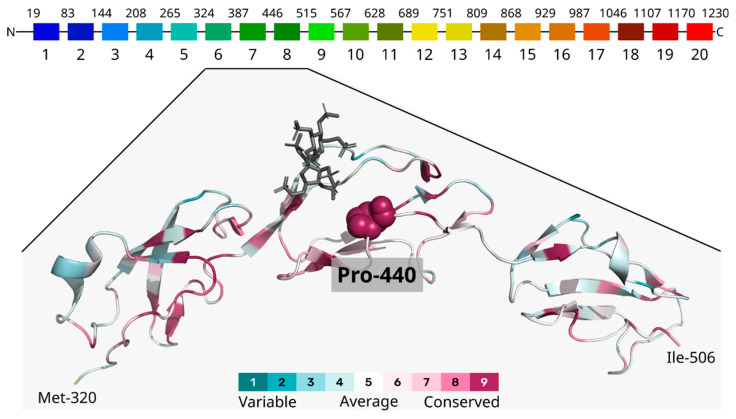
The mutated residue Pro440 is located at the C-terminus of the Sushi domain 7 of the *CFH* protein and is predicted to be conserved within the domains 6–8 (PDB structure 2UWN was used for visualization, the structure is coloured according to ConSurf scores, and the sugar ligand is grey).

The identified missense variant at position 440 of the *CFH* protein replaces proline, a non-polar, aliphatic, and structurally restricted amino acid, with serine, which is polar, neutral, and offers greater structural flexibility within the Sushi 7 domain (residues 387–444). Analysis of evolutionary conservation across species indicates that the residue at this position is conserved, as determined by ConSurf analysis (Figure 4). To investigate potential new chemical contacts resulting from the serine substitution, we compared the predicted structures of both the wild-type and mutated *CFH* proteins. The wild-type structure was sourced from the AlphaFold Database (VoroMQA global score: 0.452), while the mutated protein’s structure was predicted using AlphaFold2 via ColabFold (VoroMQA global score: 0.436)—both predictions exhibited high reliability. Applying the VoroContacts tool for the structural analyses resulted in no specific new chemical contacts when comparing proline and serine at position 440. While this residue is highly conserved evolutionarily, the AlphaMissense prediction states this substitution to be ambiguous (score of 0.405), a classification that aligns with the absence of any newly formed contacts. Notably, substitutions at the same 440 position to, for example, tryptophan, methionine, or tyrosine, are predicted by AlphaMissense to be likely pathogenic.

### 3.4. CFH Concentration in Plasma

Mean plasma *CFH* concentration in the study population was 1459 ± 212 mg/mL (min 1155 mg/mL, max 1796 mg/mL). There was no difference in *CFH* concentration in the variant carriers vs. non-carriers (1427 ± 185 mg/mL vs. 1553 ± 343 mg/mL, respectively).

### 3.5. sC5b-9 Concentration in Serum

The mean serum sC5b-9 concentration in the individuals with familial variant (proband III-6 and her relatives II-2, II-3, III-7, III-4, IV-14) was 690.7 ± 302 ng/mL (min 392 ng/mL ± 20%, max 1184 ng/mL ± 20%). These results suggest that individuals carrying the variant exhibit elevated serum sC5b-9 levels compared to the normal reference range of 200–325 ng/mL (Hedges’ g = 1.23, large). The only non-carrier adult family member’s analysis showed normal sC5b-9 concentration30—1 ng/mL ± 20%.

## 4. Discussion

Similarly to conventional hard and soft drusen, which are more commonly found in older patients with AMD, CD are located between the BL of the RPE and the inner collagenous layer of Bruch‘s membrane [34]. As documented in this case series, CD appear as multiple small elevations of the RPE in OCT scans, resulting in a typical “saw-tooth” pattern [8,9,34]. At the stage of isolated CD without complications, the disease is often asymptomatic [34], as illustrated by the proband (III-6), her younger sister (III-7), and cousin (III-4), who are currently in their 40s and maintain good BCVA.

As the disease progresses, CD become more numerous, coalesce into larger soft drusen, and later regress spontaneously, leaving areas of RPE atrophy, which may progress to geographic atrophy (GA) [5]. Other sight-threatening complications include the development of vitelliform macular detachment and CNV, typically observed in patients over 60 [5,9]. In this study, atrophy and CNV are evident in the older family members (II-3 and II-2), who are currently in their 60s and exhibit a significant decrease in BCVA.

These five family members carry the unpublished NM_000186.4(*CFH*):c.1318C>T variant. In addition, the proband’s 21-year-old daughter also carries the same variant but exhibits no signs of CD. Her young age could explain this, as the CD typically appears in individuals between the ages of 30 and 50 [11,35]. On the other hand, this genetic change was not detected in the gDNA sample of the proband’s sister III-5, who presented with a few small extrafoveal drusen in only one eye. Given these findings’ unilateral and nonprogressive nature over 7 years, there is a lack of characteristic signs of the CD disease entity. Therefore, in this case, the presence of drusen might be attributed to other environmental or endogenous risk factors [10].

CD should be distinguished from other clinical entities resulting in bilateral EODM, which appear before the age of 50 years. These diseases include large colloid drusen (LCD), familial dominant drusen (FDD), and Sorsby macular dystrophy (SMD). LCD can be distinguished clinically from CD by their large size, multiple dome-shaped RPE detachments, and distribution in the temporal portion of the macula [36]. FDD are characterized by large radial drusen, located not only in the macula, but nasally to the optic disc as well [37]. Similarly to CD, both LCD and FDD are usually asymptomatic until the development of complications, such as extensive pigmentary changes, atrophy or CNV [36,37]. In contrast, SMD, which also exhibits drusen-like deposits in the macula and along the vascular arcades, typically becomes symptomatic at a younger age, as patients can experience nyctalopia even in early stages of the disease. As the dystrophy advances, it causes severe progressive central and peripheral vision loss from around the 4th–6th decade of life [38].

In the late stages of these macular dystrophies, clinical differentiation between them and the CD subtype of AMD can become complicated, as the fibrotic and atrophic lesions resulting from different etiologies may appear similar. For this reason, genetic testing plays an important role in the differential diagnostics process. FDD results from the p.Arg345Trp mutation in the *EFEMP1* gene, which is inherited in an autosomal dominant pattern [37,39]. SMD can be caused by multiple different mutations in the *TIMP3* gene and shows autosomal dominant inheritance, as well [38]. Regarding LCD, no clear genetic associations have been identified [40]. Therefore, exclusion of mutations in the aforementioned genes may facilitate establishing an accurate diagnosis.

The *CFH* gene is localized in the 1q31.3 locus and produces two products: full-length complement factor H (FH) protein and alternative splice variant factor H-like protein (FHL-1) [41]. The transcripts encoding these two isoforms have an identical sequence of 1–9 exons and share the same N-terminal sequence of the protein (Sushi 1–7 domains).

*CFH* products play an important role in regulating the alternative pathway of the complement cascade, which is essential for clearing pathogens and immune complexes and modulating adaptive immunity [15]. The primary function of the complement system is to identify and facilitate the elimination of pathogens, waste, and dead cells by triggering the proteolytic cascade [42]. The complement system’s alternative pathway must be carefully managed to avoid excessive activation and prevent inflammation-induced damage to the body’s tissues [3,41]. FH and FHL-1 inhibit the complement system’s alternative pathway via regulating C3 levels, which is the key component in complement amplification, eventually activating the membrane attack complex (SC5b-9) [41]. By silencing the *CFH* gene in human RPE cells, Armento et al. showed that reduced levels and activity of FH lead to elevated levels of inflammatory cytokines, chemokines, and growth factors [3]. *CFH*, a key fluid-phase and surface-bound regulator of the alternative complement pathway, partially exerts its inhibitory function through interactions with glycosaminoglycans via its C-terminal domains, including Sushi 6, 7, and 8 [43,44,45].Pathogenic variants in the *CFH* gene are associated with two autosomal dominant ophthalmic phenotypes (CD (MIM#126700) and AMD, 4 (MIM#610698)) and two renal phenotypes (hemolytic uremic syndrome, atypical, susceptibility to, 1 (MIM#235400) and complement factor H deficiency (MIM#609814)) inherited in both autosomal dominant and autosomal recessive patterns [46]. Most *CFH* gene variants are disease-specific (AMD/EODM or aHUS/complement factor H deficiency), but some have a dual impact. They may be responsible for any of these phenotypes (ophthalmic and renal) in different individuals. Still, usually they are restricted to only one disorder, raising questions about the mechanism of disease pathogenesis. Although the variants causing or associated with AMD are distributed along the coding *CFH* gene sequence or splicing sites, several clustering regions were observed [47]. AMD-associated variants tend to be located in the N terminus of both protein isoforms (FH and FHL-1), mainly in Sushi1-4 and Sushi7 domains. Recently performed functional analysis proved that *CFH* insufficiency and other types of loss of protein function result in an activated complement system, leading to AMD [48]. Variants in the N-terminal region result in decreased cofactor H activity of C3b cleavage mediated by FI [49]. Earlier research by Clark et al. showed that FHL-1 can passively diffuse through the Bruch membrane, unlike the full-length FH, suggesting that FHL-1 is the primary regulator responsible for protecting the Bruch membrane from the harmful effects of complement activation [50]. There is growing evidence that complement system regulation defects result in central retinal inflammation, one of the key pathological features of AMD, leading to drusen formation, tissue injury, and vision loss [51].

The missense variant NM_000186.4(*CFH*):c.1318C>T, NP_000177.2:p.(Pro440Ser), rs761518362, identified in our family, is localized in the Sushi 7 domain, which was earlier shown to be enriched in AMD-associated variants. This protein domain acts in binding with heparan sulfates and CRP [52]. Therefore, the identified *CFH* variant might alter interactions of factor H with molecules of the intercellular space and inflammation proteins, eventually leading to activation of the alternative complement pathway. The variant has not been described in scientific literature before. It is presumed to change nonpolar amino acid proline to polar serine in position 440 of the protein. Although data in the ClinVar database and previous research demonstrate that protein-truncating variants prevail in the structure of the pathogenic/likely pathogenic variants of the *CFH* gene, many missense changes have been shown to cause AMD [47]. The replacement of proline, a rigid, structure-breaking amino acid, with serine, a more flexible polar residue, within the structurally crucial Sushi 7 domain, suggests a subtle yet significant conformational alteration. The change in amino acid properties could influence the overall tertiary structure or alter dynamics, impacting the affinity and specificity of *CFH*’s interaction with crucial GAG ligands, essential for its complement regulatory function on cell surfaces [53].

Our observations are highly relevant to macular degeneration pathogenesis, where aberrant activation of the alternative complement pathway is a well-established driver of inflammation and retinal damage [54]. Our findings align with the existing literature, emphasizing the importance of the Sushi 7 domain and the adjacent linker region in mediating *CFH* binding to GAGs [43,44]. Impaired GAG binding due to variants in the Sushi 7 region can lead to uncontrolled complement activation on retinal pigment epithelial cells and Bruch’s membrane, directly contributing to age-related macular degeneration pathology [43,44,54].

To further elucidate the impact of the identified variant, we tested *CFH* concentration in the plasma of the proband and her family members. Additionally, we quantified sC5b-9 in serum to assess the functional consequences of the variant on the activity of the membrane attack complex (MAC). Since no difference in plasma *CFH* concentration between variant carriers and non-carriers was observed in our study, we propose that the variant is associated with normal plasma levels. The results of SC5b-9 testing in the serum of adult family members revealed an elevation of MAC concentration in all carriers of the variant and a normal concentration in the non-carrier III-5 sister of the proband, which might indicate the decreased *CFH* functional activity due to the c.1318C>T variant. However, direct functional testing of *CFH*, including binding, cofactor activity, and hemolysis assays, is required to support the hypothesis.

Missense variants in plasma proteins eventually end up in two types of phenotypes. Type I variants alter protein concentration, and Type II variants impair the protein function at normal protein levels [55,56]. Therefore, genetic alterations may cause functional defects affecting protein activity, consistent with the Type II phenotype. Another possibility is that the complement system is abnormally activated locally within the eye, without detectable changes in systemic plasma levels of *CFH* [57]. While in silico analyses, particularly the AlphaMissense prediction, categorized the p.(Pro440Ser) variant as ambiguous and structure modeling did not reveal any novel specific inter-residue contacts, the evolutionary conservation and our experimental data demonstrating elevated serum C5b-9 levels indicates a functional impact on complement regulation suggesting that systemic complement activation Type II phenotype is most probably the case in this family. Earlier independent studies have also shown elevated complement sC5b-9 concentration in the blood of AMD (specifically neovascular AMD) individuals compared to controls, reflecting systemic proinflammatory complement activation [58,59]. To investigate our hypothesis further, studies involving larger cohorts and direct functional analyses of systemic and local *CFH* protein are necessary for validation.

This study has several limitations. While studying members of one family allows precise segregation analysis, providing strong evidence for causality in affected individuals, the small sample size may limit the generalizability of the findings. To confirm the results, genotype—phenotype and natural history studies in larger cohorts are needed. In addition, no in vitro functional assays were conducted to directly assess the biological impact of the NM_000186.4(*CFH*): c.1318C>T variant. While the elevated serum C5b-9 levels observed in affected family members support a functional effect on complement regulation, experimental validation is required to confirm the pathogenicity of this mutation.

## 5. Conclusions

In this study, we demonstrated the genotype–phenotype association and the natural clinical course of the disease in individuals affected with CD carrying the identified NM_000186.4(*CFH*):c.1318C>T variant. The presence of characteristic clinical signs in our study participants suggests a pathogenic role of this alteration in the development of this rare subtype of AMD.

## Data Availability

The main data generated and analyzed during this study are included in this article and its Supplementary Information Files. Any additional information is available from the authors upon request.

## Supplementary Materials

The following supporting information can be downloaded at: https://www.mdpi.com/article/10.3390/medicina61091649/s1. Figure S1: Macular OCT scans of the family members; Table S1: Virtual Ophthalmic Panel genes; Table S2: Primers for PCR of the *CFH* gene exon 9 and Sanger sequencing title.

## Abbreviations

The following abbreviations are used in this manuscript:

AMD	Age-related macular degeneration
anti-VEGF	Vascular endothelial growth factor inhibitors
BL	Basal lamina
BCVA	Best corrected visual acuity
CD	Cuticular drusen
CNV	Choroidal neovascularization
DFE	Dilated fundus examination
EODM	Early-onset drusen maculopathy
FDD	Familial dominant drusen
GA	Geographic atrophy
IRF	Intraretinal fluid
gDNA	Genomic DNA
LCD	Large colloid drusen
LE	Left eye
MAC	Membrane attack complex
nAMD	Neovascular AMD
OCT	Optical coherence tomography
OD	Optical density
PCR	Polymerase chain reaction
RE	Right eye
RPE	Retinal pigment epithelium
SMD	Sorsby macular dystrophy
SRF	Subretinal fluid
VUHSK	Vilnius University Hospital Santaros Klinikos
WES	Whole-exome sequencing

## References

[1] Sergejeva O., Botov R., Liutkevičienė R., Kriaučiūnienė L. (2016). Genetic factors associated with the development of age-related macular degeneration. Medicina.

[2] Wong W.L., Su X., Li X., Cheung C.M.G., Klein R., Cheng C.Y., Wong T.Y. (2014). Global prevalence of age-related macular degeneration and disease burden projection for 2020 and 2040: A systematic review and meta-analysis. Lancet Glob. Health.

[3] Armento A., Schmidt T.L., Sonntag I., Merle D.A., Jarboui M.A., Kilger E., Clark S.J., Ueffing M. (2021). *CFH* Loss in Human RPE Cells Leads to Inflammation and Complement System Dysregulation via the NF-κB Pathway. Int. J. Mol. Sci..

[4] Taylor R.L., Poulter J.A., Downes S.M., McKibbin M., Khan K.N., Inglehearn C.F., Webster A.R., Hardcastle A.J., Michaelides M., Bishop P.N. (2019). Loss-of-Function Mutations in the *CFH* Gene Affecting Alternatively Encoded Factor H-like 1 Protein Cause Dominant Early-Onset Macular Drusen. Ophthalmology.

[5] Fragiotta S., Fernández-Avellaneda P., Breazzano M.P., Scuderi G. (2021). Clinical Manifestations of Cuticular Drusen: Current Perspectives. Clin. Ophthalmol..

[6] Guigui B., Leveziel N., Martinet V., Massamba N., Sterkers M., Coscas G., Souied E.H. (2011). Angiography features of early onset drusen. Br. J. Ophthalmol..

[7] Boon C.J., van de Ven J.P., Hoyng C.B., Hollander A.I.D., Klevering B.J. (2013). Cuticular drusen: Stars in the sky. Prog. Retin. Eye Res..

[8] van de Ven J.P., Boon C.J., Smailhodzic D., Lechanteur Y.T., Hollander A.I.D., Hoyng C.B., Theelen T. (2012). Short-Term Changes of Basal Laminar Drusen on Spectral-Domain Optical Coherence Tomography. Am. J. Ophthalmol..

[9] Shin D.H., Kong M., Han G., Han J.C., Ham D.I. (2020). Clinical manifestations of cuticular drusen in Korean patients. Sci. Rep..

[10] Thomas C.J., Mirza R.G., Gill M.K. (2021). Age-Related Macular Degeneration. Med. Clin. N. Am..

[11] Boon C.J., Klevering B.J., Hoyng C.B., Zonneveld-Vrieling M.N., Nabuurs S.B., Blokland E., Cremers F.P., Hollander A.I.D. (2008). Basal Laminar Drusen Caused by Compound Heterozygous Variants in the *CFH* Gene. Am. J. Hum. Genet..

[12] Fritsche L.G., Igl W., Bailey J.N.C., Grassmann F., Sengupta S., Bragg-Gresham J.L., Burdon K.P., Hebbring S.J., Wen C., Gorski M. (2016). A large genome-wide association study of age-related macular degeneration highlights contributions of rare and common variants. Nat. Genet..

[13] Deng Y., Qiao L., Du M., Qu C., Wan L., Li J., Huang L. (2022). Age-related macular degeneration: Epidemiology, genetics, pathophysiology, diagnosis, and targeted therapy. Genes Dis..

[14] Grassi M.A., Folk J.C., Scheetz T.E., Taylor C.M., Sheffield V.C., Stone E.M. (2007). Complement Factor H Polymorphism p.Tyr402His and Cuticular Drusen. Arch. Ophthalmol..

[15] Duvvari M.R., van de Ven J.P.H., Geerlings M.J., Saksens N.T.M., Bakker B., Henkes A., Neveling K., del Rosario M., Westra D., Heuvel L.P.W.J.v.D. (2016). Whole Exome Sequencing in Patients with the Cuticular Drusen Subtype of Age-Related Macular Degeneration. PLoS ONE.

[16] Merle D.A., Sen M., Armento A., Stanton C.M., Thee E.F., Meester-Smoor M.A., Kaiser M., Clark S.J., Klaver C.C., Keane P.A. (2023). 10q26—The enigma in age-related macular degeneration. Prog. Retin. Eye Res..

[17] Sambrook J., Russell D.W. (2006). Purification of nucleic acids by extraction with phenol:chloroform. CSH Protoc..

[18] Jiang H., Lei R., Ding S.W., Zhu S. (2014). Skewer: A fast and accurate adapter trimmer for next-generation sequencing paired-end reads. BMC Bioinform..

[19] Li H., Durbin R. (2009). Fast and accurate short read alignment with Burrows–Wheeler transform. Bioinformatics.

[20] Wang K., Li M., Hakonarson H. (2010). ANNOVAR: Functional annotation of genetic variants from high-throughput sequencing data. Nucleic. Acids Res..

[21] Richards S., Aziz N., Bale S., Bick D., Das S., Gastier-Foster J., Grody W.W., Hegde M., Lyon E., Spector E. (2015). Standards and guidelines for the interpretation of sequence variants: A joint consensus recommendation of the American College of Medical Genetics and Genomics and the Association for Molecular Pathology. Genet. Med..

[22] Robinson J.T., Thorvaldsdóttir H., Winckler W., Guttman M., Lander E.S., Getz G., Mesirov J.P. (2011). Integrative genomics viewer. Nat. Biotechnol..

[23] Ye J., Coulouris G., Zaretskaya I., Cutcutache I., Rozen S., Madden T.L. (2012). Primer-BLAST: A tool to design target-specific primers for polymerase chain reaction. BMC Bioinform..

[24] Varadi M., Bertoni D., Magana P., Paramval U., Pidruchna I., Radhakrishnan M., Tsenkov M., Nair S., Mirdita M., Yeo J. (2023). AlphaFold Protein Structure Database in 2024: Providing structure coverage for over 214 million protein sequences. Nucleic. Acids Res..

[25] Jumper J., Evans R., Pritzel A., Green T., Figurnov M., Ronneberger O., Tunyasuvunakool K., Bates R., Žídek A., Potapenko A. (2021). Highly accurate protein structure prediction with AlphaFold. Nature.

[26] Mirdita M., Schütze K., Moriwaki Y., Heo L., Ovchinnikov S., Steinegger M. (2022). ColabFold: Making protein folding accessible to all. Nat. Methods.

[27] Olechnovič K., Venclovas Č. (2019). VoroMQA web server for assessing three-dimensional structures of proteins and protein complexes. Nucleic. Acids Res..

[28] Olechnovič K., Venclovas Č. (2021). VoroContacts: A Tool for the Analysis of Interatomic Contacts in Macromolecular Structures. Bioinformatics.

[29] Dapkūnas J., Timinskas A., Olechnovič K., Tomkuvienė M., Venclovas Č. (2024). PPI3D: A web server for searching, analyzing and modeling protein-protein, protein-peptide and protein-nucleic acid interactions. Nucleic. Acids Res..

[30] Ben Chorin A., Masrati G., Kessel A., Narunsky A., Sprinzak J., Lahav S., Ashkenazy H., Ben-Tal N. (2020). ConSurf-DB: An accessible repository for the evolutionary conservation patterns of the majority of PDB proteins. Protein Sci..

[31] Yariv B., Yariv E., Kessel A., Masrati G., Ben Chorin A., Martz E., Mayrose I., Pupko T., Ben-Tal N. (2023). Using evolutionary data to make sense of macromolecules with a “face-lifted” ConSurf. Protein Sci..

[32] Cheng J., Novati G., Pan J., Bycroft C., Žemgulytė A., Applebaum T., Pritzel A., Wong L.H., Zielinski M., Sargeant T. (2023). Accurate proteome-wide missense variant effect prediction with AlphaMissense. Science.

[33] R Core Team (2024). R: A Language and Environment for Statistical Computing. R Foundation for Statistical Computing. https://www.R-project.org/.

[34] Balaratnasingam C., Cherepanoff S., Dolz-Marco R., Killingsworth M., Chen F.K., Mendis R., Mrejen S., Too L.K., Gal-Or O., Curcio C.A. (2018). Cuticular Drusen. Ophthalmology.

[35] Goh K.L., Chen F.K., Balaratnasingam C., Abbott C.J., Hodgson L.A., Guymer R.H., Wu Z. (2022). Cuticular Drusen in Age-Related Macular Degeneration. Ophthalmology.

[36] Roberti N.C., Dias JRde O., Novais E.A., Regatieri C.S., Belfort R. (2017). Large colloid drusen analyzed with structural en face optical coherence tomography. Arq. Bras. Oftalmol..

[37] Zhang K., Sun X., Chen Y., Zhong Q., Lin L., Gao Y., Hong F. (2018). Doyne honeycomb retinal dystrophy/malattia leventinese induced by EFEMP1 mutation in a Chinese family. BMC Ophthalmol..

[38] Anand-Apte B., Chao J.R., Singh R., Stöhr H. (2019). Sorsby Fundus Dystrophy: Insights from the past and looking to the future. J. Neurosci. Res..

[39] Fu L., Garland D., Yang Z., Shukla D., Rajendran A., Pearson E., Stone E.M., Zhang K., Pierce E.A. (2007). The R345W mutation in EFEMP1 is pathogenic and causes AMD-like deposits in mice. Hum. Mol. Genetics..

[40] Khan K.N., Mahroo O.A., Khan R.S., Mohamed M.D., McKibbin M., Bird A., Michaelides M., Tufail A., Moore A.T. (2016). Differentiating drusen: Drusen and drusen-like appearances associated with ageing, age-related macular degeneration, inherited eye disease and other pathological processes. Prog. Retin. Eye Res..

[41] Armento A., Ueffing M., Clark S.J. (2021). The complement system in age-related macular degeneration. Cell. Mol. Life Sci..

[42] Ricklin D., Hajishengallis G., Yang K., Lambris J.D. (2010). Complement: A key system for immune surveillance and homeostasis. Nat. Immunol..

[43] Herbert A.P., Deakin J.A., Schmidt C.Q., Blaum B.S., Egan C., Ferreira V.P., Pangburn M.K., Lyon M., Uhrín D., Barlow P.N. (2007). Structure shows that a glycosaminoglycan and protein recognition site in factor H is perturbed by age-related macular degeneration-linked single nucleotide polymorphism. J. Biol. Chem..

[44] Prosser B.E., Johnson S., Roversi P., Herbert A.P., Blaum B.S., Tyrrell J., Jowitt T.A., Clark S.J., Tarelli E., Uhriín D. (2007). Structural basis for complement factor H–linked age-related macular degeneration. J. Exp. Med..

[45] Schneider M.C., Prosser B.E., Caesar J.J.E., Kugelberg E., Li S., Zhang Q., Quoraishi S., Lovett J.E., Deane J.E., Sim R.B. (2009). Neisseria meningitidis recruits factor H using protein mimicry of host carbohydrates. Nature.

[46] Online Mendelian Inheritance in Man, OMIM^®^. McKusick-Nathans Institute of Genetic Medicine, Johns Hopkins University (Baltimore, MD). https://omim.org/.

[47] Geerlings M.J., Volokhina E.B., de Jong E.K., Van De Kar N., Pauper M., Hoyng C.B., van den Heuvel L.P., den Hollander A.I. (2018). Genotype-Phenotype Correlations of Low-Frequency Variants in the Complement SYSTEM in renal Disease and Age-Related Macular Degeneration. Clin. Genet..

[48] *CFH* Haploinsufficiency and Complement Alterations in Early-Onset Macular Degeneration IOVS ARVO Journals. https://iovs.arvojournals.org/article.aspx?articleid=2793613.

[49] Geerlings M.J., Kremlitzka M., Bakker B., Nilsson S.C., Saksens N.T., Lechanteur Y.T., Pauper M., Corominas J., Fauser S., Hoyng C.B. (2017). The Functional Effect of Rare Variants in Complement Genes on C3b Degradation in Patients with Age-Related Macular Degeneration. JAMA Ophthalmol..

[50] Clark S.J., Schmidt C.Q., White A.M., Hakobyan S., Morgan B.P., Bishop P.N. (2014). Identification of Factor H–like Protein 1 as the Predominant Complement Regulator in Bruch’s Membrane: Implications for Age-Related Macular Degeneration. J. Immunol..

[51] McHarg S., Clark S.J., Day A.J., Bishop P.N. (2015). Age-related macular degeneration and the role of the complement system. Mol. Immunol..

[52] Giannakis E., Jokiranta T.S., Male D.A., Ranganathan S., Ormsby R.J., Fischetti V.A., Mold C., Gordon D.L. (2003). A common site within factor H SCR 7 responsible for binding heparin, C-reactive protein and streptococcal M protein. Eur. J. Immunol..

[53] Blackmore T.K., Sadlon T.A., Ward H.M., Lublin D.M., Gordon D.L. (1996). Identification of a heparin binding domain in the seventh short consensus repeat of complement factor H. J. Immunol..

[54] Toomey C.B., Pflugmacher S., Park K., Pihl J., Novak S.W., Rodriguez J., Jalali M., Jung J., Mozafari M., Omran S.P. (2025). Bruch’s membrane heparan sulfate retains lipoproteins in the early stages of age-related macular degeneration. Proc. Natl. Acad. Sci. USA.

[55] Rodriguez E., Rallapalli P.M., Osborne A.J., Perkins S.J. (2014). New functional and structural insights from updated mutational databases for complement factor H, Factor I, membrane cofactor protein and C3. Biosci. Rep..

[56] Saunders R.E., Abarrategui-Garrido C., Frémeaux-Bacchi V., de Jorge E.G., Goodship T.H., Trascasa M.L., Noris M., Castro I.M.P., Remuzzi G., de Córdoba S.R. (2007). The interactive Factor H-atypical hemolytic uremic syndrome mutation database and website: Update and integration of membrane cofactor protein and Factor I mutations with structural models. Hum. Mutat..

[57] Warwick A., Khandhadia S., Ennis S., Lotery A. (2014). Age-Related Macular Degeneration: A Disease of Systemic or Local Complement Dysregulation?. J. Clin. Med..

[58] Lynch A.M., Mandava N., Patnaik J.L., A Frazer-Abel A., Wagner B.D., Palestine A.G., Mathias M.T., Siringo F.S., Cathcart J.N., Holers V.M. (2020). Systemic activation of the complement system in patients with advanced age-related macular degeneration. Eur. J. Ophthalmol..

[59] Kijlstra A., Berendschot T.T.J.M. (2015). Age-related macular degeneration: A complementopathy?. Ophthalmic Res..

