# Supplementary material for: Evidence for the Pathogenicity of a CFH Variant in a Multigenerational Family with Cuticular Drusen
Source: Medicina (Kaunas). 2025 Sep 11;61(9):1649. doi: 10.3390/medicina61091649 (PMC12472062; doi:10.3390/medicina61091649)
Supplement: Supplementary file 1 [file medicina-61-01649-s001.zip › medicina-3811516-supplementary/Figure S1.docx]

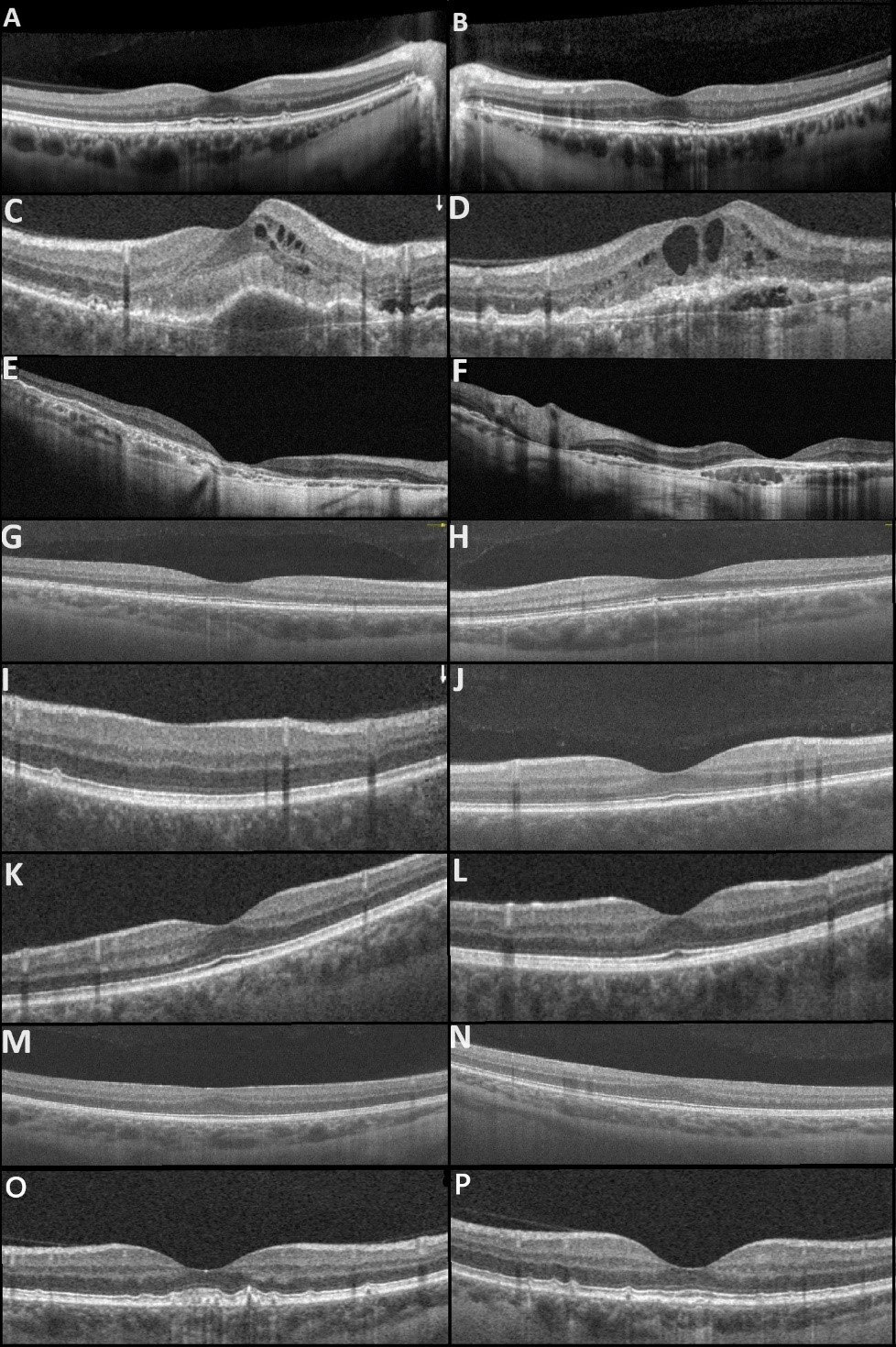


**Figure S1.  Macular OCT scans of the family members.** The proband‘s (III-6) OCT scans of the RE (A) and LE (B) show numerous small subfoveal RPE elevations, characteristic of CD. Scans C and D show the maculae of the RE and LE of the proband’s father (II-3) upon diagnosis. Subfoveal fibrosis, IRF cysts, and multiple extrafoveal CD are visible. Images E (right eye) and F (left eye) show the maculae of the proband’s paternal aunt after treatment discontinuation. Atrophy of the RPE and photoreceptors and a fibrotic scar in the left eye are visible. Images G and H show several CDs without any complications in the maculae of the proband’s sister III-7. Image I reveals an isolated extrafoveal druse in the RE of the proband’s other sister (III-5). In contrast, image J shows no pathological changes in the same patient's LE. Images K and L show healthy maculae of the proband’s daughter (IV-14), while M and N show fovea plana configuration of the proband’s son (IV-15) with no signs of drusen. Images O (right eye) and P (left eye) show the CD of family member III-4.
