# Supplementary material for: Evidence for the Pathogenicity of a CFH Variant in a Multigenerational Family with Cuticular Drusen
Source: Medicina (Kaunas). 2025 Sep 11;61(9):1649. doi: 10.3390/medicina61091649 (PMC12472062; doi:10.3390/medicina61091649)
Supplement: Supplementary file 1 [file medicina-61-01649-s001.zip › medicina-3811516-supplementary/Table S1.docx]

Supplementary Table S1. Virtual Ophthalmic Panel genes.

| *ABCA4* | *ARL6* | *C2* | *CYP27A1* | *CRYBB1* | *EYA1* | *FOXE3* | *GUCA1B* | *INVS* | *LRMDA* | *MT-TH* | *PAK2* | *PLK4* | *RBP4* | *SCLT1* | *SPTLC2* | *TOGARAM1* | *WFS1* |
| --- | --- | --- | --- | --- | --- | --- | --- | --- | --- | --- | --- | --- | --- | --- | --- | --- | --- |
| *ABCB6* | *ARMC9* | *C21orf2* | *CYP4V2* | *CRYBB2* | *EIF2B2* | *FOXI2* | *GUCY2D* | *IQCB1* | *LRP2* | *MT-TL1* | *PANK2* | *PLOD3* | *RCBTB1* | *SDCCAG8* | *SRD5A3* | *TOPORS* | *WHRN* |
| *ABCC6* | *ARMS2* | *C2CD3* | *CYP51A1* | *CRYBB3* | *EYS* | *FOXL2* | *HARS* | *IQSEC2* | *LRP5* | *MTTP* | *PANK4* | *PMM2* | *RD3* | *SDHA* | *SREBF1* | *TPP1* | *WLS* |
| *ABCD1* | *ARR3* | *C2orf71* | *CISD2* | *CRYGB* | *ELOVL1* | *FRAS1* | *HARS1* | *IRX5* | *LRRC32* | *MT-TP* | *PAX2* | *PNKP* | *RDH11* | *SEC23A* | *SSBP1* | *TRAF3IP1* | *WNT3* |
| *ABHD12* | *ARSG* | *C3* | *CLCC1* | *CRYGC* | *ELOVL4* | *FREM1* | *HCCS* | *ISCA2* | *LSS* | *MT-TS2* | *PAX3* | *PNPLA6* | *RDH12* | *SEMA3E* | *STRA6* | *TREX1* | *WRN* |
| *ACBD5* | *ASB10* | *C5orf42* | *CLCN7* | *CRYGD* | *ELP4* | *FREM2* | *HDAC6* | *ITM2B* | *LTBP2* | *MVK* | *PAX6* | *POC1B* | *RDH5* | *SEMA4A* | *STX3* | *TRIM32* | *XYLT2* |
| *ACO2* | *ASCC3L1* | *C8orf37* | *CLDN19* | *CRYGS* | *EMC1* | *FRMD7* | *HDAC8* | *ITPA* | *LZTFL1* | *NAA10* | *PCARE* | *POC5* | *RDS* | *SERAC1* | *SUFU* | *TRIM44* | *XPA* |
| *ACOX1* | *ASPH* | *C9* | *CLEC3B* | *CRX* | *EP300* | *FSCN2* | *HESX1* | *ITPR1* | *MAB21L2* | *NACC1* | *PCDH15* | *POLG* | *RECQL4* | *SETX* | *SUMF1* | *TRNT1* | *ZEB1* |
| *ACTB* | *ATAD3A* | *CA4* | *CLN3* | *CSPP1* | *EPG5* | *FTL* | *HEXA* | *JAG1* | *MAF* | *NBAS* | *PCYT1A* | *POLG2* | *REEP6* | *SH3PXD2B* | *SUOX* | *TRPM1* | *ZEB2* |
| *ACTG1* | *ATF6* | *CABP4* | *CLN5* | *CTC1* | *EPHA2* | *FUT5* | *HGSNAT* | *JAM3* | *MAFB* | *NDP* | *PDE6A* | *POLH* | *RERE* | *SHH* | *TACSTD2* | *TSFM* | *ZFYVE26* |
| *ADAM9* | *ATG7* | *CACNA1A* | *CLN6* | *CTDP1* | *EPRS* | *FXN* | *HHAT* | *KAT6B* | *MAG* | *NDUFA12* | *PDE6B* | *POLR1C* | *RGR* | *SIL1* | *TBC1D20* | *TSPAN12* | *ZIC2* |
| *ADAMTS10* | *ATOH7* | *CACNA1F* | *CLN8* | *CTNNA1* | *ERCC1* | *FZD4* | *HIKESHI* | *KCNJ13* | *MAK* | *NDUFB11* | *PDE6C* | *POLR1D* | *RGS9* | *SIPA1L3* | *TBC1D23* | *TTC21B* | *ZNF408* |
| *ADAMTS17* | *ATP13A2* | *CACNA2D4* | *CLPB* | *CTNNB1* | *ERCC2* | *FZD5* | *HYLS1* | *KCNV2* | *MAN2B1* | *NDUFS1* | *PDE6D* | *POMGNT1* | *RGS9BP* | *SIX3* | *TCF4* | *TTC8* | *ZNF423* |
| *ADAMTS18* | *ATXN7* | *CANT1* | *CLRN1* | *CTNND1* | *ERCC3* | *GALK1* | *HK1* | *KCTD7* | *MAPKAPK3* | *NDUFS2* | *PDE6G* | *POMGNT2* | *RHO* | *SIX5* | *TCIRG1* | *TTLL5* | *ZNF469* |
| *ADAMTSL4* | *ATXN7* | *CAPN15* | *CLUAP1* | *CTSD* | *ERCC4* | *GALM* | *HKDC1* | *KDM6A* | *MAPRE2* | *NDUFS3* | *PDE6H* | *POMK* | *RHOA* | *SIX6* | *TCOF1* | *TTPA* | *ZNF513* |
| *ADGRA3* | *AUH* | *CAPN5* | *CNBP* | *CWC27* | *ERCC5* | *GALT* | *HMCN1* | *KERA* | *MC1R* | *NEK2* | *PDSS1* | *POMT1* | *RIC1* | *SLC16A12* | *TCTN1* | *TUB* | *ZNF526* |
| *ADGRV1* | *B3GALNT2* | *CBS* | *CNGA1* | *CX3CR1* | *ERCC6* | *GBA* | *HMGB3* | *KIAA0556* | *MCAT* | *NF2* | *PDZD7* | *POMT2* | *RIMS1* | *SLC24A1* | *TCTN2* | *TUBA3D* | *ZNF644* |
| *ADIPOR1* | *B3GLCT* | *CC2D2A* | *CNGA3* | *DAG1* | *ERCC8* | *GCNT2* | *HMX1* | *KIAA0586* | *MCOLN1* | *NHS* | *PEX1* | *PORCN* | *RIMS2* | *SLC24A5* | *TCTN3* | *TUBB* | *ZNHIT3* |
| *AFG3L2* | *B4GAT1* | *CCDC28B* | *CNGB1* | *DAGLA* | *ESCO2* | *GDF3* | *HOXA1* | *KIAA0753* | *MECR* | *NYX* | *PEX10* | *PPT1* | *RIPK4* | *SLC25A24* | *TDRD7* | *TUBB2B* | *ZPR1* |
| *AGBL1* | *B9D1* | *CDH2* | *CNGB3* | *DCC* | *ESPN* | *GDF6* | *HPS1* | *KIAA1109* | *MED12* | *NLRP3* | *PEX11B* | *PQBP1* | *RYR1* | *SLC25A4* | *TEAD1* | *TUBB3* |  |
| *AGBL5* | *B9D2* | *CDH23* | *CNNM4* | *DCN* | *EVR3* | *GEMIN4* | *HPS3* | *KIAA1549* | *MED27* | *NMNAT1* | *PEX12* | *PRCD* | *RLBP1* | *SLC25A46* | *TEK* | *TUBB4B* |  |
| *AGK* | *BBIP1* | *CDH3* | *COG4* | *DCT* | *EXOC8* | *GFER* | *HPS4* | *KIF11* | *MERTK* | *NPHP1* | *PEX13* | *PRDM13* | *RNASEH1* | *SLC2A1* | *TENM3* | *TUBGCP4* |  |
| *AHI1* | *BBS1* | *CDHR1* | *COL11A1* | *DDB1* | *EXOSC2* | *GJA1* | *HPS5* | *KIF21A* | *MFF* | *NPHP3* | *PEX14* | *PRDM5* | *RNLS* | *SLC33A1* | *TFAP2A* | *TUBGCP6* |  |
| *AHR* | *BBS10* | *CDK9* | *COL11A2* | *DDX58* | *FAM111A* | *GJA3* | *HPS6* | *KIF3B* | *MFN2* | *NPHP4* | *PEX16* | *PRKCG* | *RNPC3* | *SLC38A8* | *TFG* | *TULP1* |  |
| *AIPL1* | *BBS12* | *CDON* | *COL17A1* | *DDX59* | *FAM126A* | *GJA8* | *HSD17B4* | *KIF7* | *MFRP* | *NR2E3* | *PEX19* | *PROM1* | *RNU4ATAC* | *SLC44A1* | *TGFBI* | *TWNK* |  |
| *AIRE* | *BBS2* | *CENPF* | *COL18A1* | *DGUOK* | *FAM149B1* | *GLI2* | *HSF4* | *KIT* | *MFSD6L* | *NR2F1* | *PEX2* | *PRPF3* | *ROBO3* | *SLC45A2* | *TIMM8A* | *UBIAD1* |  |
| *AKR1E2* | *BBS4* | *CEP104* | *COL25A1* | *DHCR7* | *FAM161A* | *GLI3* | *HTRA1* | *KIZ* | *MFSD8* | *NRL* | *PEX26* | *PRPF31* | *ROM1* | *SLC4A11* | *TYMP* | *UCHL1* |  |
| *ALDH18A1* | *BBS5* | *CEP120* | *COL2A1* | *DHDDS* | *FAM57B* | *GLS* | *HTRA2* | *KLHL7* | *MGME1* | *NSUN2* | *PEX3* | *PRPF4* | *RP1* | *SLC4A4* | *TIMP3* | *UNC119* |  |
| *ALDH1A3* | *BBS7* | *CEP164* | *COL4A1* | *DHX38* | *FANCA* | *GMPPB* | *YAP1* | *KMT2D* | *MYH9* | *NTF4* | *PEX5* | *PRPF6* | *RP1L1* | *SLC52A2* | *TINF2* | *UNC45B* |  |
| *ALDH3A2* | *BBS9* | *CEP19* | *COL5A1* | *DKC1* | *FANCD2* | *GNAT1* | *IDH3A* | *KRT12* | *MYO5A* | *NUP188* | *PEX6* | *PRPF8* | *RP2* | *SLC6A6* | *TYR* | *USH1C* |  |
| *ALG3* | *BCOR* | *CEP250* | *COL8A2* | *DMD* | *FANCE* | *GNAT2* | *IDH3B* | *KRT3* | *MYO7A* | *OAT* | *PEX7* | *PRPH2* | *RP9* | *SLC7A14* | *TYRP1* | *USH1G* |  |
| *ALMS1* | *BEST1* | *CEP290* | *COL9A1* | *DMPK* | *FANCI* | *GNB3* | *IFIH1* | *LAMA1* | *MYOC* | *OCA2* | *PGK1* | *PRPS1* | *RPE65* | *SLC9A6* | *TK2* | *USH1J* |  |
| *ALPK1* | *BFSP1* | *CEP41* | *COL9A2* | *DNA2* | *FANCL* | *GNPAT* | *IFT140* | *LAMB2* | *MIP* | *OCRL* | *PHF6* | *PRR12* | *RPGR* | *SMAD4* | *TKFC* | *USH2A* |  |
| *ALX1* | *BFSP2* | *CEP78* | *COL9A3* | *DNAJC19* | *FAR1* | *GNPTAB* | *IFT172* | *LAMP2* | *MIPEP* | *OFD1* | *PHGDH* | *PRSS56* | *RPGRIP1* | *SMCHD1* | *TMEM107* | *USH3B* |  |
| *ALX3* | *BLOC1S3* | *CERKL* | *COPB1* | *DNAJC30* | *FAT1* | *GNPTG* | *IFT27* | *LARGE1* | *MITF* | *OPA1* | *PHYH* | *PSMC3* | *RPGRIP1L* | *SMG9* | *TMEM126A* | *USP45* |  |
| *AMACR* | *BLOC1S6* | *CFAP410* | *COQ2* | *DNM1L* | *FBLN5* | *GP1BA* | *IFT74* | *LCA5* | *MKKS* | *OPA2* | *PHOX2A* | *PTCH1* | *RRM2B* | *SMO* | *TMEM138* | *VAX1* |  |
| *ANAPC1* | *BMP4* | *CFB* | *COQ4* | *DNMBP* | *FBN1* | *GPR143* | *IFT81* | *LCAT* | *MKS1* | *OPA3* | *PIBF1* | *PUF60* | *RS1* | *SMOC1* | *TMEM216* | *VCAN* |  |
| *ANTXR1* | *BMP7* | *CFH* | *COQ5* | *DOCK6* | *FDXR* | *GPR179* | *IGBP1* | *LEMD2* | *MMACHC* | *OPA4* | *PIGL* | *PXDN* | *RTN4IP1* | *SNRNP200* | *TMEM218* | *VIM* |  |
| *AP3B1* | *BRPF1* | *CFI* | *COX7B* | *DPYD* | *FGFR1* | *GPR98* | *IKBKG* | *LETM1* | *MPDZ* | *OPN1LW* | *PIK3C2A* | *RAB18* | *SAG* | *SNX10* | *TMEM231* | *VPS13B* |  |
| *AP3B2* | *BUB1B* | *CHD7* | *CPAMD8* | *DRAM2* | *FYCO1* | *GRHL2* | *YME1L1* | *LIG3* | *MPLKIP* | *OPN1MW* | *PIK3R1* | *RAB28* | *SALL1* | *SNX3* | *TMEM237* | *VPS4A* |  |
| *AP3D1* | *C10ORF11* | *CHM* | *CPLANE1* | *DTHD1* | *FKRP* | *GRIP1* | *IMPDH1* | *LIM2* | *MSMO1* | *OPN1SW* | *PIK3R5* | *RAB3GAP1* | *SALL2* | *SOX10* | *TMEM5* | *VSX1* |  |
| *APOA1* | *C10orf2* | *CHMP4B* | *CRB1* | *DTNBP1* | *FKTN* | *GRK1* | *IMPG1* | *LYST* | *MSTO1* | *OPTN* | *PIKFYVE* | *RAB3GAP2* | *SALL4* | *SOX2* | *TMEM67* | *VSX2* |  |
| *APTX* | *C12orf57* | *CHN1* | *CREBBP* | *EDN3* | *FLVCR1* | *GRM6* | *IMPG2* | *LMX1B* | *MT-ATP6* | *OSTM1* | *PISD* | *RARB* | *SAMD11* | *SPATA7* | *TMEM70* | *VWA8* |  |
| *ARHGEF18* | *C12orf65* | *CHRDL1* | *CRYAA* | *EDNRB* | *FNBP4* | *GRN* | *INPP5E* | *LONP1* | *MT-ND1* | *OTX2* | *PITPNM3* | *RAX* | *SAMD7* | *SPG7* | *TMEM98* | *WDPCP* |  |
| *ARL13B* | *C16orf62* | *CHST6* | *CRYAB* | *EED* | *FOSL2* | *GSN* | *INPP5K* | *LOXL1* | *MT-ND4* | *OVOL2* | *PITX2* | *RAX2* | *SBF2* | *SPINT2* | *TMX3* | *WDR19* |  |
| *ARL2BP* | *C19orf12* | *CIB2* | *CRYBA1* | *EFEMP1* | *FOXC1* | *GTF2H5* | *INTS1* | *LRAT* | *MT-ND6* | *P3H2* | *PITX3* | *RB1* | *SC5D* | *SPP2* | *TNFRSF11A* | *WDR36* |  |
| *ARL3* | *C1QTNF5* | *CYP1B1* | *CRYBA4* | *EFTUD2* | *FOXD3* | *GUCA1A* | *INTS8* | *LRIT3* | *MTPAP* | *PACS1* | *PLA2G5* | *RBP3* | *SCAPER* | *SPTLC1* | *TNFSF11* | *WDR37* |  |
