# Supplementary material for: Evidence for the Pathogenicity of a CFH Variant in a Multigenerational Family with Cuticular Drusen
Source: Medicina (Kaunas). 2025 Sep 11;61(9):1649. doi: 10.3390/medicina61091649 (PMC12472062; doi:10.3390/medicina61091649)
Supplement: Supplementary file 1 [file medicina-61-01649-s001.zip › medicina-3811516-supplementary/Table S2.docx]

Supplementary Table S2. Primers for PCR of the *CFH* gene exon 9 and Sanger sequencing.

| No | Primer | Sequence | T_m_(°C) | Amplicon length(bp) |
| --- | --- | --- | --- | --- |
| 1 | *CFH*_9F | GAGTTGTTCAAGCAAAGTGACCA | 59.87 | 635 |
| 2 | *CFH*_9R | GTCCATTGGTAAAACAAGGTGACAT | 59.99 |  |
